# Autoimmunity in Primary Immunodeficiency Disorders: An Updated Review on Pathogenic and Clinical Implications

**DOI:** 10.3390/jcm10204729

**Published:** 2021-10-15

**Authors:** Giorgio Costagliola, Susanna Cappelli, Rita Consolini

**Affiliations:** Section of Clinical and Laboratory Immunology, Division of Pediatrics, Department of Clinical and Experimental Medicine, University of Pisa, 56126 Pisa, Italy; giorgio.costagliola@hotmail.com (G.C.); susicappelli82@yahoo.it (S.C.)

**Keywords:** 22q11.2 deletion syndrome, activated phosphoinositide 3-kinase d syndrome, common variable immunodeficiency, complement deficiency, CTLA-4, Immune dysregulation, LRBA, selective IgA deficiency, severe combined immunodeficiency, X-linked agammaglobulinemia

## Abstract

During the last years, studies investigating the intriguing association between immunodeficiency and autoimmunity led to the discovery of new monogenic disorders, the improvement in the knowledge of the pathogenesis of autoimmunity, and the introduction of targeted treatments. Autoimmunity is observed with particular frequency in patients with primary antibody deficiencies, such as common variable immunodeficiency (CVID) and selective IgA deficiency, but combined immunodeficiency disorders (CIDs) and disorders of innate immunity have also been associated with autoimmunity. Among CIDs, the highest incidence of autoimmunity is described in patients with autoimmune polyendocrine syndrome 1, LRBA, and CTLA-4 deficiency, and in patients with STAT-related disorders. The pathogenesis of autoimmunity in patients with immunodeficiency is far to be fully elucidated. However, altered germ center reactions, impaired central and peripheral lymphocyte negative selection, uncontrolled lymphocyte proliferation, ineffective cytoskeletal function, innate immune defects, and defective clearance of the infectious agents play an important role. In this paper, we review the main immunodeficiencies associated with autoimmunity, focusing on the pathogenic mechanisms responsible for autoimmunity in each condition and on the therapeutic strategies. Moreover, we provide a diagnostic algorithm for the diagnosis of PIDs in patients with autoimmunity.

## 1. Introduction

In recent years, the association between primary immunodeficiency disorders (PIDs) and autoimmunity has been extensively studied. Patients with PIDs can develop an immune dysregulation of variable degree, which is responsible for a clinical picture featured by infectious complications and autoimmunity [1,2]. Autoimmune manifestations are observed with considerable frequency in patients with primary antibody deficiencies, including common variable immunodeficiency (CVID) and selective IgA deficiency (sIgAD), but can also be evidenced in patients with combined immunodeficiency disorders (CID) [3]. Notably, autoimmunity can represent the presentation sign of PIDs in a significant number of patients [1]. The molecular mechanisms responsible for the immune dysregulation in patients with PIDs are multiple and not completely elucidated; impaired B cell differentiation and germ-center reactions, altered T cell central or peripheral tolerance, uncontrolled lymphocyte proliferation and differentiation, dysfunctional complement, and innate immune activation can participate in the complex pathogenic process leading to autoimmunity. In patients with PIDs, the association with autoimmunity leads to a significant impact on the quality of life, higher medicalization, and increased mortality [2]. Furthermore, the increasing use of new sequencing techniques allowed the identification of different monogenic causes of PID, the better understanding of genotype-phenotype correlations, and the improvement of the therapeutic strategies targeting the immune dysregulation in PIDs [4,5].

In this paper, we review the main autoimmune manifestations observed in patients with PIDs, focusing on the molecular mechanisms implicated in the pathogenesis of the immune imbalance in each condition. Furthermore, we provide some key issues for the diagnostic and therapeutic approach to autoimmunity in patients suffering from PIDs.

## 2. Autoimmunity in Primary Antibody Deficiency Disorders

Among primary antibody deficiencies, autoimmunity has been described with considerable frequency in patients with CVID, sIgAD, and hyper-IgM syndrome (HIGM), with the clinical phenotype being significantly associated with the variability of the genetic background (Table 1).

### 2.1. Common Variable Immunodeficiency

CVID is characterized by a reduction in serum immunoglobulin (Ig) G and IgA, by at least two standard deviations below age-appropriate reference levels, with or without low Ig M levels, accompanied by poor antibody response to vaccines or low switched-memory B cells. Clinical diagnosis of CVID requires at least one between increased susceptibility to infections, autoimmune manifestations, granulomatous disease, lymphoproliferation, or an affected family member with antibody deficiency [6]. A total of 30–50% of CVID patients have non-infectious manifestations, including autoimmune, gastrointestinal, pulmonary, lymphoproliferative, and malignant complications [7,8,9], which strongly contribute to morbidity and mortality [7,10]. Autoimmune diseases occur in 20–30% of CVID patients. Autoimmune cytopenias, and in particular immune thrombocytopenic purpura (ITP), autoimmune hemolytic anemia (AIHA), and Evans syndrome [9,11], are the most commonly reported, but organ-specific and systemic autoimmune diseases are also described. Interestingly, cytopenia may be the first manifestation of the immune defect, in patients without a typical history of infections [12]. Concerning systemic autoimmune diseases, in a recent study of 870 CVID patients from the United States Immunodeficiency Network (USIDNET) registry, 5% were found to have a rheumatologic disease [13]. The most common rheumatologic manifestation reported in CVID is inflammatory arthritis, occurring in about 3% of patients, but systemic lupus erythematosus (SLE), Sjögren’s disease, Behçet disease, and psoriasis have also been described [10,13,14,15]. Among organ-specific autoimmune diseases, in a European Society of Immune Deficiencies (ESID) registry of 2700 CVID patients, hypothyroidism was the most prevalent at 3.5%, followed by alopecia areata, vitiligo, and type I diabetes (T1D) [9]. Among autoimmune manifestations, only cytopenias have been associated with decreased survival and CVID-associated noninfectious complications, including lymphoproliferation, granulomatous disease, lymphoma, hepatic disease, pulmonary involvement (granulomatous lymphocytic interstitial lung disease (GLILD)), and enteropathy [7].

Different studies suggested that both genetic background and immunological abnormalities play a significant role in explaining the link between CVID and autoimmunity. CVID patients can show deregulated immune responses at different levels, involving altered germ center reactions, class switch, and B cell proliferation [11]. Additionally, an impaired suppressive function of Bregs on activated T cells leading to excessive T cell activation has been described [16]. T cell dysfunction is a contributing factor in the development of autoimmunity in CVID [17]. Hyperactivated T cell phenotype [18], reduced number and function of regulatory T cells (Tregs) [19], and an increase in T helper type 1 (TH1), type 17 (TH17), and T helper follicular cells have been observed in CVID patients with autoimmunity [11,20,21]. Moreover, autoimmunity may be caused not only by a break in tolerance to self-antigens but also by the inability of CVID patients to completely eradicate microbial antigens, resulting in compensatory, often exaggerated, and chronic inflammatory responses [2].

Even if many CVID patients may have a polygenic disease, in about 15–30% of CVID cases, a monogenic cause has been found [22]. CVID patients with *TNFRSF13B* mutation (encoding for the B cell-activating factor (BAFF) and APRIL receptor, TACI), especially if heterozygous, have a propensity to autoimmune manifestations and lymphoid hyperplasia potentially due to lack of normal mechanisms required to establish tolerance [23]. *BAFF-R* mutations, which may impair B-cell maturation, have also been described in association with autoimmunity [24,25]. Autoimmunity and other clinical manifestations (including lymphoproliferation) have been associated with the deficiency of *NF-kB1* and *NF-kB2*, which are transcription factors that are crucial for B-cell maturation, survival, differentiation, class switching, and self-tolerance. Additionally, it is described in patients with mutations affecting the inducible T-cell co-stimulator (*ICOS*), a T cell surface receptor that is closely related to NF-kB activation and is essential for terminal B cell differentiation and immune tolerance [25]. Finally, autoimmunity has been described in patients with mutations in other genes implicated in B cell activation and proliferation, including PLCγ2, which is responsible for the PLCγ2-associated antibody deficiency and immune dysregulation (PLAID) [25,26].

### 2.2. Selective IgA Deficiency

sIgAD is defined, according to ESID and the International Union of Immunological Societies (IUIS), as serum levels of <7 mg/dL in individuals older than 4 years in the presence of normal levels of both IgG and IgM, normal IgG antibody response to vaccinations and exclusion of other causes of hypogammaglobulinemia and T-cell defects [6]. Although most of the patients with sIgAD are asymptomatic, some patients develop various clinical manifestations, such as minor recurrent sinopulmonary infections, allergies, and autoimmune manifestations [27]. A variety of autoimmune diseases may be overrepresented in patients with sIgAD than the normal population and sometimes autoimmunity could be the only clinical manifestation in these patients [27]. The prevalence of autoimmune disorders in patients with sIgAD varies from 5 to 30% [28,29,30,31,32,33,34,35,36,37,38,39,40,41,42,43,44,45,46,47,48,49,50,51,52,53,54,55,56,57,58,59,60,61,62,63,64,65,66,67,68,69,70,71,72,73,74,75,76,77,78,79,80], with celiac disease, ITP, AIHA, autoimmune thyroiditis, T1D, RA, and SLE being the most frequently observed manifestations [28,29,31,32].

Several mechanisms have been suggested in the development of autoimmunity in sIgAD [32], including the association with specific HLA haplotypes (particularly, the haplotype 8.1) [33], T and B cells or cytokine abnormalities, shared genetic susceptibility, or ineffective antigen clearance with molecular mimicry. Concerning immune dysfunction, Tregs deficiency is observed in 64% of the patients [34], and a lower number of CD4 + lymphocytes and switched memory B cells have been described in patients with sIgA [35]. Additionally, it has been observed that sIgAD patients with a lower number of switched memory B cells are more prone to infections and autoimmunity [30]. The monogenic hypothesis suggests that certain monogenic mutations predispose both to the development of sIgAD and autoimmune diseases. Interestingly, similar variants of CTLA4-ICOS have been found in celiac disease, sIgAD, and CVID [36]. Functionally, as IgA protect mucosal barriers from the entry of foreign antigens, in patients with sIgAD, pathogens can easily penetrate the mucosa and through a mechanism of molecular mimicry and cross-reaction with self-antigens might cause the formation of self-reactive antibodies [29,37]. Additionally, the lack of IgA may cause defective removal of immune complexes, thus propagating a state of persistent local and systemic inflammation, which may predispose to the sensitization of immune cells to self-antigen s [29]. Finally, IgA interact with cell receptors (as FcαRI) to downregulate immune pathways and protect against autoimmunity, and this function is impaired in patients with sIgAD [29].

### 2.3. Hyper IgM Syndrome

The HIGM syndromes are a group of primary immunodeficiency disorders in which defective Ig class switch recombination, with or without defects of somatic hypermutation, leads to deficiency of IgG, IgA, and IgE with preserved or elevated levels of IgM [38]. Among HIGM syndromes, there is a genetic heterogeneity supported by the existence of X-linked, autosomal recessive, and autosomal dominant inheritance. Among X-linked HIGM (XHIM), the most common form is caused by mutations in the gene encoding CD40 ligand (CD40L) a molecule transiently expressed on the surface of activated T cells [39]. Mutations of NEMO/IKKγ genes are implicated in the X-linked Anhidrotic Ectodermal Dysplasia with Immunodeficiency (EDA-ID), a syndrome associated with HIGM. Mutations of NEMO lead to an abnormal expression of multiple enzymes required for antibody switching, such as activation-induced cytidine deaminase (AIG) and uracil DNA glycosylase (UNG), while mutations in IKKγ gene result in blockage of NF-kB release into the nucleus interfering with NF-kB and downstream CD40 signaling [40]. Concerning autosomal recessive HIGM, mutations in the AID and UNG genes result in HIGM syndrome with pure humoral immunodeficiency associated with lymphoid hypertrophy [41,42]. The natural receptor of CD40L is CD40, which is expressed on antigen-presenting cells, including B cells (APCs), dendritic cells, and macrophages. Additionally, mutations in the CD40 gene have been described in patients with HIGM, who present a very similar clinical picture to boys with XHIM [43]. In addition to susceptibility to infections, HIGM patients are prone to develop autoimmune diseases, in particular those with mutations in CD40L, CD40, AID, and NEMO [44]. The prevalence of autoimmune manifestations in X-linked HIGM has been reported to be 10–20% without considering neutropenia, whose etiology is not well understood, with seronegative arthritis, thyroiditis, and SLE being the most commonly observed manifestations [45,46]. Regarding recessive HIGM, autoimmunity is described in about 20% of the patients with AID deficiency, and the manifestations consist of AIHA, ITP, hepatitis, T1D, Chron’s disease, and uveitis [47]. The mechanisms responsible for autoimmunity in HIGM are heterogeneous and depend on the genetic background. In the XHIM form, the defective CD40-CD40L-mediated interaction results in the failure of elimination of self-reactive B cells, reduction of Tregs [48], and altered cytokine secretion [49], while in the recessive form, AID deficiency could result in defective regulation of self-reactive B cells [50].

### 2.4. X-Linked Agammaglobulinemia

X-linked agammaglobulinemia (XLA) is caused by a B lymphocyte differentiation arrest caused by mutations in the BTK gene, and is predominantly featured by recurrent infections (especially with encapsulated bacterial pathogens) caused by antibody deficiency with nearly undetectable levels of peripheral B cells [51]. XLA patients appear to be at an increased risk of developing autoimmune diseases, which can be found in up to 15% of patients [52]. Arthritis is the most frequent autoimmune presentation of XLA patients [52] but dermatomyositis, inflammatory bowel diseases (IBD), AIHA, scleroderma, alopecia, T1D, and glomerulonephritis have also been described [53,54,55]. Chronic inflammation due to subclinical infections significantly contributes to immune dysregulation in XLA patients. Evidence supports the notion that BKT-dependent, but antibody-independent, mechanisms may be involved in the pathophysiology of autoimmunity in XLA [56]. Excessive stimulation by pathogen molecules of Toll-like receptors (TLRs) may contribute to inducing autoimmunity. Indeed, by certain mutations of BTK and recurrent infections, in XLA patients, overstimulation of TLR9 and its secondary messengers, NF-kB, may occur [57], thus causing enhanced production of autoantibodies from innate B-1 cells [58].

### 2.5. Therapeutic Approach to Autoimmunity in Primary Antibody Deficiencies

The treatment strategies of autoimmune manifestations in patients with PADS are generally the same as in immune-competent patients and include the use of high dose intravenous immunoglobulins (IVIg) and immunosuppressive agents, such as corticosteroids, methotrexate, and azathioprine (resulting in an increased risk of infections). As a second-line therapy, rituximab appears to be highly effective and relatively safe for the management of severe immune cytopenias [59]. On the other hand, splenectomy is reserved as a last resource in patients who have failed all other therapies [60] and is generally disfavored because of the risk of subsequent infections.

## 3. Autoimmunity in Severe Combined Immunodeficiency and Related Disorders

The genetic and clinical variability among severe combined immunodeficiency (SCID) is remarkable and in some patients, the phenotype of early-onset severe infections can be associated with autoimmunity [61]. The case of Omenn syndrome (OS) is of particular interest, since in this condition the immune impairment coexists with a marked tendency towards lymphoproliferation and autoimmunity [62]. Children with OS develop severe invasive infections in the first months of life, and commonly show hepatosplenomegaly, diffuse lymphadenopathy, severe eczema, and alopecia [63]. Laboratory testing evidence peripheral eosinophilia, lymphopenia, and reduced serum immunoglobulin levels, associated with the peripheral expansion of self-reactive T cells, which represents the most peculiar aspect of OS [64].

The molecular basis underlying this clinical phenotype relies in most of the patients on a mutation of the recombinase activating genes (RAG) 1 and 2, which are central in the V(D)J recombination during T and B cell development. However, defects in other proteins (such as IL-7Ra, ZAP70, ARTEMIS, AK2, JAK3, and others) can be responsible for OS [61]. The pathogenesis of the autoimmune phenotype is not completely elucidated, but defects in central negative selection secondary to reduced AIRE expression and altered peripheral tolerance are implicated [65]. The prognosis of children with OS is severe [64], with death occurring in the first years of life unless they receive definitive treatment with HSCT [66]. The clinical expression of RAG mutations is not limited to OS. Mutations causing a partial loss of function of RAG cause an extremely variable clinical phenotype, with a wide spectrum of severity and clinical features of combined immunodeficiency, immune dysregulation with autoimmunity (mainly autoimmune cytopenia), and lymphoproliferation [62,67,68].

Among the non-OS phenotypic variants of SCID, patients with ARTEMIS deficiency show an ineffective DNA repair with genomic instability, with a consequent clinical picture of SCID associated with radiosensitivity and immune dysregulation with autoimmunity. Autoimmunity has also been described in a few cases of SCID carrying other molecular defects (i.e., IL-7Ra, ZAP70, ADA, and PNP deficiency) as the result of an altered central negative selection of T-cells [61]. Finally, mutations in the ORAI1 and STIM1 genes, encoding for calcium channels implicated in multiple cell functions (including B-cell receptor (BCR) and T-cell receptor (TCR) signaling), are responsible for a clinical phenotype featured by SCID-like manifestations, autoimmunity, hypotonia, and ectodermal dysplasia [65]. In all these conditions, HSCT represents the only curative therapeutic strategy.

## 4. Autoimmunity in Disorders of T-Cell Central Tolerance

The immunologic tolerance for T cells is reached through the process of central negative selection of self-reactive T cell progenitors, the peripheral induction of anergy of cells escaping central tolerance, and the action of Tregs [69]. Autoimmune polyendocrine syndrome (APS-1) and 22q11.2 deletion syndrome (22q11.2DS) represent two paradigmatic examples of autoimmunity caused by impaired central negative selection of T cells.

### 4.1. Autoimmune Polyendocrine Syndrome 1

APS-1 is a rare monogenic disorder with autosomal recessive inheritance caused by mutations altering the function of the AIRE gene, expressed by thymic medullary cells and thymic dendritic cells (DCs) [70]. AIRE promotes the production of a wide range of proteins expressed in other tissues, thus causing their presentation to immature thymocytes and driving the process of central negative selection. Moreover, AIRE induces the production of Tregs, contributing to the elimination of self-reactive T cells [71]. As a consequence, defective AIRE function is associated with an expansion of self-reactive lymphocytes and the production of different specificities of autoantibodies, with high variability among APS-1 patients. Although there are no specific autoantibodies allowing the diagnosis of APS-1, a significant percentage of patients exhibit antibodies directed against cytokines implicated in the immune and inflammatory response, such as interferon (IFN), IL-17, and IL-22, contributing to the immune impairment and dysregulation observed in this disease [71]. The main clinical features of APS-1 are represented by chronic mucocutaneous candidiasis, Addison’s disease, and primary hypoparathyroidism, but the phenotypic spectrum comprehends autoimmune enteropathy, hepatitis, pancreatitis, nephritis, and other clinical manifestations [70,71]. Treatment strategies are not uniformed, since they are strongly influenced by the prominent clinical manifestations observed in the individual patient [72]. Indeed, while endocrine complications are treated with hormonal replacement therapy, autoimmune organ involvement often requires the use of steroids, immunosuppressive agents (such as azathioprine), or rituximab [70].

### 4.2. 22q11.2 Deletion Syndrome

The wide clinical spectrum of 22q11.2DS, also known as DiGeorge syndrome (DGS), comprehends both congenital abnormalities (cardiac malformation, velo/palatal dysfunction, parathyroid insufficiency) and immunological alterations [73]. Children with 22q11.2DS show variable severity of immune impairment, ranging from complete athymia to different degrees of combined immunodeficiency with reduced thymic function (low levels of naïve T cells and recent emigrants T (RTE) cells) and increased risk of autoimmunity, which is observed in about 10% of the patients [74,75]. The autoimmune manifestations more commonly evidenced in 22q11.2DS are ITP, juvenile idiopathic arthritis (JIA), and thyroiditis. Additionally, enteropathy and cutaneous autoimmunity (alopecia, psoriasis, vitiligo) have been described [76].

**Figure 1:**
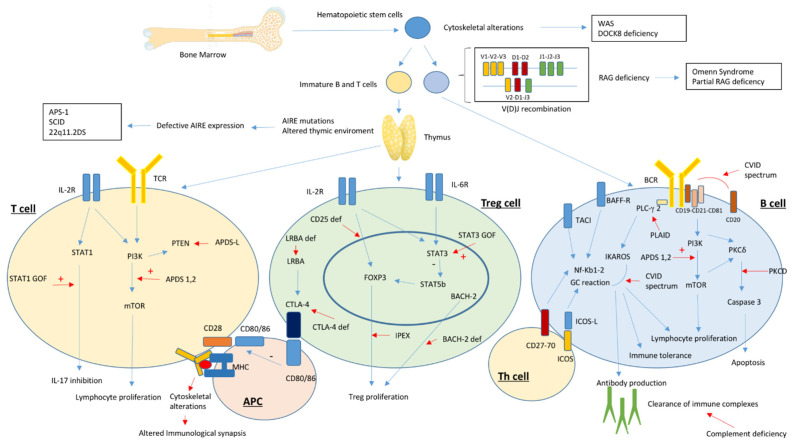
Pathogenesis of autoimmunity in immunodeficiency disorders. Figure legend: 22q11.2DS: chromosome 22q11.2 deletion syndrome; APC: antigen-presenting cells; APDS: Activated phosphoinositide 3-kinase d syndrome; APS-1: Autoimmune polyendocrine syndrome 1; CTLA-4: Cytotoxic lymphocyte antigen 4; CVID: Common variable immunodeficiency; LRBA: LPS-responsive beige-like anchor protein; PI3K: Phosphoinositide 3-kinase; PKCD: protein kinase C δ deficiency; PKCδ: protein kinase C δ; RAG: Recombinase activating genes; SCID: severe combined immunodeficiency; STAT: Signal Transducers and Activator of Transcription; WAS: Wiskott-Aldrich syndrome; XLA: X-linked agammaglobulinemia.

In 22q11.2DS, the pathogenesis of autoimmunity involves multiple mechanisms. The abnormal thymic environment [77] is associated with reduced expression of AIRE (Figure 1), thus impairing T cell negative selection, and reduced generation of Tregs [65,74,75,78]. Moreover, the ineffective immune response, with consequent persistence of microbial antigens, could lead to the phenomenon of molecular mimicry [2]. Recently, alterations in DCs subpopulations have also been described in 22q11.2DS, with reduced circulating numbers of both myeloid DCs (mDCs) and plasmacytoid DCs [79]. This could contribute to the development of autoimmunity, since pDCs have an important role in maintaining peripheral immune tolerance [79]. Interestingly, while the infectious phenotype is prevalent during early childhood, autoimmunity is commonly observed at a higher age. This partly reflects an evolution of the immunological phenotype of 22q11.2DS patients, with progressive reduction of Tregs and expansion of self-reactive T cells [79,80].

When the immune function is preserved and patients experience a low rate of infections, the use of corticosteroids and conventional immunosuppressive agents represents the initial therapeutic strategy to treat autoimmunity, while the therapeutic approach is more blurred in patients with severe infectious complications [73].

## 5. Autoimmunity in Disorders of T-Cell Peripheral Tolerance

Among disorders of peripheral tolerance, conditions affecting Treg function (also called “Tregopathies”) have a prominent relevance [3]. The most frequent disorder of Tregs is the immune dysregulation, polyendocrinopathy, enteropathy, X-linked (IPEX) syndrome, which is featured by a classic clinical triad of eczema, enteropathy, and endocrine autoimmunity, without a significant increase in infectious morbidity [81]. Recent genetic advances allowed the identification of different monogenic disorders featured by an IPEX-like clinical phenotype accompanied with increased susceptibility to infections, placing them at a molecular, pathogenic, and clinical crossroad between immunodeficiency and autoimmunity [81,82].

### 5.1. CTLA-4 Deficiency

Cytotoxic lymphocyte antigen 4 (CTLA-4) is a molecule expressed by Treg cells that has a central role in the induction of peripheral immune tolerance. Indeed, CTLA-4 reduces the expression of CD80 and CD86 on the surface of antigen-presenting cells. As CD80 and CD86 are essential proteins for the costimulatory signal in the immunologic synapsis between APC and T cells, their depletion causes a reduced activation of T cells and differentiation in effector cells [83].

Patients with CTLA-4 deficiency show a picture of combined immunodeficiency with lymphopenia (reduced naïve T cells, Tregs, CD19 cells), hypogammaglobulinemia, and susceptibility to viral and bacterial infections accompanied by a high rate of autoimmune and lymphoproliferative manifestations [2,83]. The autoimmune spectrum observed in CTLA-4 deficiency is variable, comprehending autoimmune cytopenia, arthritis, uveitis, endocrinopathies, and enteropathy. In this condition, lymphoproliferation is observed in about 50% of the patients and presenting with lymphadenopathy, hepatosplenomegaly, and, although in a reduced percentage of patients, with pulmonary involvement, in the form of GLILD [62,84]. In patients with CTLA-4 deficiency, surveillance for the risk of lymphomas is essential, and treatment of autoimmunity and lymphoproliferation comprehends the use of sirolimus and the biologic agent abatacept, a fusion molecule containing the extracellular domain of CTLA-4 [85].

### 5.2. LRBA Deficiency

LPS-responsive beige-like anchor protein (LRBA) is a protein implicated in intracellular trafficking, which acts by inhibiting the lysosome degradation of CTLA-4 [86]. Therefore, it is essential for maintaining adequate expression of CTLA-4 on the cellular surface, and its deficiency shares several common features with CTLA-4 deficiency. Indeed, patients with LRBA deficiency often present with recurrent sinopulmonary infections, hepatosplenomegaly, lymphadenopathy, and autoimmune cytopenia [87,88]. Among the other autoimmune manifestations observed in this condition, there are enteropathy, endocrinopathies (thyroiditis, T1D), hepatitis, and uveitis [89]. Therefore, patients can present with both an IPEX-like and a CVID-like clinical phenotype, and the immunological assessment commonly shows hypogammaglobulinemia and lymphopenia, with a reduced absolute number of Tregs and memory B cells [87]. Among classic immunosuppressive strategies, there is interest in the role of sirolimus and hydroxychloroquine, as this drug can potentially reduce CTLA-4 degradation. Although it has shown promising results on autoimmune manifestations and immune abnormalities, there are only a few reports of patients treated with abatacept and, similarly, the experience with HSCT in this condition is still limited [85,90,91].

### 5.3. STAT-Related Disorders

The family of Signal Transducers and Activator of Transcription (STAT) molecules is involved in multiple signaling pathways activated by different cytokines and controls the transcription of genes implicated in the immune and inflammatory response [92]. In particular, the activation of STAT-1 is mostly mediated by IFN-α and IL-2, while STAT-3 is also influenced by IL-6. The molecular mechanism leading to STAT phosphorylation requires the presence of Janus kinase (JAK) molecules [92]. Impaired or enhanced function of the JAK/STAT-dependent molecular pathways can result in a wide spectrum of immunological and clinical alterations, with immune dysregulation and susceptibility to infections being the most relevant features.

In STAT1 gain of function (GOF), patients show reduced proliferation of TH17 cells, causing increased susceptibility to different infections, and typically present with chronic mucocutaneous candidiasis [93]. Moreover, up to a third of the patients develop autoimmune manifestations, that are mainly represented by endocrinopathies, autoimmune cytopenia, and enteropathy [81].

STAT-3 GOF is also featured by an increased risk of severe infections, deriving from a combined immune defect, associated with a high incidence of autoimmunity (cytopenia, enteropathy, endocrinopathy, arthritis). In this condition, patients also frequently display lymphoproliferation with hepato-splenomegaly [94]. The molecular defect underlying this phenotype involves reduced Tregs and Th17 proliferation [95].

Finally, STAT5b deficiency causes impaired IL-2 signaling, with consequently reduced proliferation of Tregs. The disease presents with a picture of combined immunodeficiency, growth hormone insensitivity, and IPEX-like immune dysregulation [81,96].

**Figure 2:**
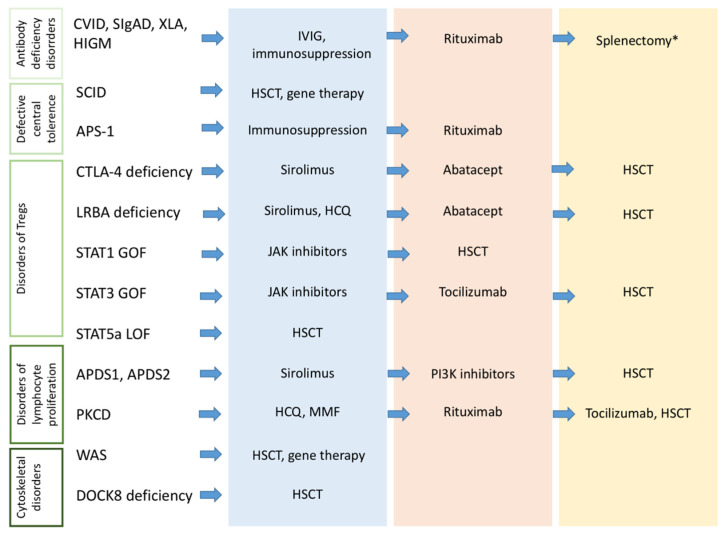
Therapeutic strategies for autoimmunity in patients with PIDs. The figure shows the current therapeutic options for specific PIDs. The choice of the therapeutic strategy (immunosuppressive agents, biologic drugs, HSCT, gene therapy) depends on the clinical severity, comorbidities and also on the availability and physician’s experience. * In patients with refractory autoimmune cytopenia. APDS: Activated phosphoinositide 3-kinase d syndrome; APS-1: Autoimmune polyendocrine syndrome 1; CTLA-4: Cytotoxic lymphocyte antigen 4; CVID: Common variable immunodeficiency; HCQ: Hydroxychloroquine; HIGM: Hyper-IgM syndromes; HSCT: hematopoietic stem cell transplantation; JAK: Janus kinase; LRBA: LPS-responsive beige-like anchor protein; MMF: mycophenolate mofetil; PI3K: Phosphoinositide 3-kinase; PKCD: protein kinase C δ deficiency; SCID: severe combined immunodeficiency; sIgAD: selective IgA deficiency; STAT: Signal Transducers and Activator of Transcription; Tregs: regulatory T cells; WAS: Wiskott-Aldrich syndrome; XLA: X-linked agammaglobulinemia.

Although the definitive treatment for STAT-related disorders is currently represented by HSCT, the use of JAK inhibitors has demonstrated promising responses in the management of autoimmunity, infections, and lymphoproliferation in STAT1 GOF and STAT3 GOF [85]. Additionally, given the role of IL-6 in the activation of STAT-3, the anti-IL-6 antibody tocilizumab is a promising alternative for this condition [85,97] (Figure 2).

### 5.4. Other Disorders of Regulatory T Cells

Recently, other molecular defects impairing Treg function have been described, each in a reduced number of patients. In CD25 (IL-2RA) deficiency, IL-2 signaling is significantly impaired, thus resulting in an IPEX-like clinical picture associated with infections, and lymphoproliferation [81]. The haploinsufficiency of the BACH2 transcription factor causes altered gem center reactions, reduced Tregs levels, and increased Th1 cell proliferation, finally causing hypogammaglobulinemia, sinopulmonary infections, enteropathy, and lymphoproliferation [98].

## 6. Autoimmunity in Disorders of Lymphocyte Differentiation and Proliferation

Altered lymphocyte proliferation and differentiation can be responsible for a heterogeneous range of clinical manifestations, ranging from severe infectious diseases to increased susceptibility to autoimmunity and lymphoproliferation. This pathogenic aspect has particular relevance in determining the clinical phenotype of the activated phosphoinositide 3-kinase d syndrome (APDS) and protein kinase C δ deficiency (PKCD).

### 6.1. Activated Phosphoinositide 3-Kinase d Syndrome

APDS is a combined immunodeficiency disorder associated with an increased risk of sinusitis, respiratory infections, severe herpesvirus infections, and a high rate of autoimmunity and lymphoproliferation. Indeed, autoimmune and lymphoproliferative manifestations (diffuse lymphadenopathy, hepatomegaly, splenomegaly) are the presenting sign in more than half of the patients diagnosed with APDS [99,100]. Autoimmunity occurs in about two-thirds of the patients with APDS, and the clinical expression of autoimmunity consists mostly of the finding of autoimmune cytopenia, arthritis, and enteropathy [101]. The disease is caused by mutations affecting the phosphoinositide 3-kinase (PI3K) molecular complex, which is involved in numerous signaling pathways activated after the binding of TCR and BCR with their ligands and influences cellular metabolism, proliferation, and differentiation of B and T lymphocytes [102] (Figure 1). Patients with GOF mutations in the phosphatidylinositol-4,5-bisphosphate 3-kinase Catalytic Subunit δ (PIK3CD) gene are classified as affected by APDS1, while loss of function mutation in the Phosphoinositide-3-Kinase Regulatory Subunit 1 (PIK3R1) is diagnostic for APDS2 [101]. These two molecular defects are responsible for the uncontrolled activation of the PI3K-dependent molecular pathways, including the intracellular events linked to the activation of mTOR. In APDS, a shift in cellular metabolic and proliferative activity is observed, and patients usually display a peculiar immunological phenotype, featured by high levels of senescent T lymphocytes, effector memory T cells, progressive B lymphocytopenia, and reduced absolute numbers of naïve T cells [101]. The serum levels of immunoglobulin are extremely variable, since patients can present with a CVID-like phenotype, an HIGM picture, or in some cases, hypergammaglobulinemia [99].

A correct diagnosis of APDS is mandatory since it significantly influences the therapeutic approach and the follow-up. Concerning follow-up, the surveillance against the development of lymphoid neoplasms is a central feature, since patients with APDS have an increased risk of developing lymphomas and, particularly, non-Hodgkin’s lymphomas [62,101]. Treatment of APDS comprehends the immunoglobulin replacement therapy, when necessary, and the measures to control autoimmunity and lymphoproliferation. To this point, sirolimus, an mTOR inhibitor, is commonly used as a first-line strategy, while selective PI3K inhibitors (leniolisib, nemiralisib) are given in refractory cases [101]. Finally, patients with APDS could benefit from HSCT, although there is no uniform consensus on the timing of the transplantation and the conditioning regimen [103].

### 6.2. Protein Kinase C δ Deficiency

Mutation impairing the protein kinase C δ (PKCδ) structure or function leads to a clinical phenotype featured by an increased risk of infections with hypogammaglobulinemia, autoimmunity, and lymphoproliferation [104]. PKCδ is implicated in the activation of different transcriptional factors with a central role in the immune homeostasis of B lymphocytes [105]. It is activated in the context of a wide number of molecular pathways, including mTOR, PI3K, and mediates the transcription of STAT1 (leading to enhanced IFN-stimulated transcription), ERK1, and other factors. PKCδ induces also the transcription of the IL-10 gene and reduces the production of IL-6. Finally, it promotes apoptosis through the interaction with caspase 3, thus limiting lymphocyte proliferation [104,106]. In PKCD, autoimmunity depends on the accumulation of self-reactive lymphocytes, the production of different autoantibodies, and defects in the process of lymphocyte negative selection [105]. The most common manifestations of autoimmunity are represented by arthritis, cytopenia, and SLE-like manifestations, such as glomerulonephritis and photosensitive rash [104]. Moreover, the ineffective control of apoptosis causes lymphoproliferation, and some patients presented with a clinical phenotype mimicking autoimmune lymphoproliferative syndrome (ALPS) [107]. As literature reports only a small number of cases of patients diagnosed with PKCD, the therapeutic approach has yet to be defined. However, available data suggest the use of conventional immunosuppressive strategies approved for SLE, including hydroxychloroquine, mycophenolate mofetil, and rituximab, and, when the lymphoproliferative aspect is prominent, the adoption of anti-mTOR drugs, such as sirolimus [104]. The administration of tocilizumab in patients with high IL-6 serum levels and the use of HSCT represent promising therapeutic strategies [104].

Interestingly, a similar clinical picture can be observed in the RAS-associated autoimmune leukoproliferative disease (RALD), a disease caused by mutations in the RAS signaling pathway (often NRAS or KRAS), which is currently classified among the phenocopies of PIDs. In RALD, uncontrolled activation of RAS-dependent molecular signaling is responsible for the development of hepatosplenomegaly, lymphadenopathy, autoimmune cytopenia, and SLE-like manifestations [108].

## 7. Autoimmunity in Disorders of Cytoskeletal Function

Cytoskeletal proteins are essential for multiple cellular functions, including the immunologic synapsis between T cells and APCs, and the regulation of lymphocyte proliferation.

Wiskott-Aldrich syndrome (WAS) is an inherited condition caused by mutations in the WASP gene on the X chromosome, which encodes the WAS protein, an actin-nucleation promoting factor expressed in hematopoietic stem cells [109]. WAS is clinically featured by a classic triad of thrombocytopenia with small-size platelets, eczema, and lymphopenia, mainly affecting T cells [110]. The molecular defect influences multiple cellular lineages and causes complex implications on the immune function, including ineffective T cell proliferation and function, reduced Treg activity (with preserved absolute Treg values), and hyperproliferation of B cells, which show enhanced production of autoantibodies [110]. The disease severity of WAS patients is variable, and the more commonly reported autoimmune manifestations are AIHA, autoimmune neutropenia, peripheral vasculitis, and arthritis [111]. Although patients with a clinical picture dominated by thrombocytopenia could benefit from splenectomy, this intervention does not reduce the risk of autoimmunity. Consequently, the definitive treatment of patients with WAS is currently represented by HSCT or gene therapy [111].

Another disease featured by altered cytoskeletal structure and function is DOCK8 deficiency, which shares some common clinical features with WAS. In this condition, the molecular defect is responsible for an altered cytoskeletal actin regulation, which causes reduced proliferation, migration, and function of innate and adaptive immune cells, and impaired Treg activity [112].

Clinically, patients with DOCK8 deficiency show a picture of combined immunodeficiency featured by recurrent cutaneous and respiratory infections, eczema, increased risk of malignancies, and predisposition to the development of atopy (with high IgE levels), and autoimmunity (cytopenia, thyroiditis, vasculitis, uveitis). In absence of specific gene therapy, the only curative treatment for DOCK8 deficiency is HSCT [111,112].

## 8. Autoimmunity in Complement Deficiencies and Disorders of Innate Immunity

Genetic defects causing deficiency of components of the complement cascade are associated with an increased risk of bacterial infections caused by capsulate agents. This derives from the pivotal role of the classic and alternate complement pathways in determining bacterial lysis. However, deficiencies of specific complement proteins (C1q, C1r/s, C2, C4a, C4b) also represent a risk factor for the development of different autoimmune manifestations, such as SLE-like features, glomerulonephritis, JIA, and dermatomyositis [2,113,114]. The pathogenic mechanism linking complement deficiencies with autoimmunity is mostly dependent on a reduced clearance of apoptotic cells and immune complexes. This causes an enhanced availability of auto-antigens, which are chronically exposed to the immune system, thus triggering a self-reactive immune response [114,115]. Moreover, complement deficiency can impair B-cell negative selection, thus allowing the expansion of self-reactive B-lymphocytes, and can contribute to the development of autoimmunity [113]. Since in patients with complement deficiency the immunization against capsulated bacteria significantly improves the outcome, recognizing a patient with this condition and characterizing the specific defect is essential to improve the long-term management, including the anti-infectious prophylaxis.

Concerning disorders of innate immunity, the incidence of autoimmune manifestations is lower compared to PIDs affecting the adaptive response. However, in patients with chronic granulomatous disease (CGD), a higher incidence of SLE-like clinical features, arthritis, and other autoimmune conditions (hepatitis, nephritis) is observed [116]. Although the pathogenesis of these manifestations is not completely elucidated, it is accepted that the process involves the persistence of infectious antigens, inflammasome overactivation, altered production of neutrophil extracellular traps, and defective apoptosis [2,117]. The treatment of autoimmunity in CGD is challenging, since the need to use steroids and immunosuppressive agents should be balanced with the high infectious risk observed in this population. Currently, HSCT and gene therapy are the only curative treatments for CGD [116,117].

## 9. From Theory to Bedside

The above-discussed pathogenic and clinical associations carry different significant implications for the approach to children with autoimmunity, highlighting that it should represent a warning sign for the presence of a PID, particularly in pediatric age. Identifying a condition of PID in children presenting with autoimmunity offers the opportunity to provide an adequate treatment of the underlying disease (Ig replacement therapy, targeted treatments, HSCT, gene therapy), to offer supplementary immunization if needed, and optimize the management of autoimmunity itself (Figure 2). On the other hand, as different studies have demonstrated a reduced survival in the subgroup of patients with PIDs showing autoimmunity, its finding in patients with already diagnosed PID could lead to significant changes in the follow-up strategy and therapeutic approach [118,119].

### 9.1. Diagnosing PIDs in Children Presenting with Autoimmunity

**Figure 3:**
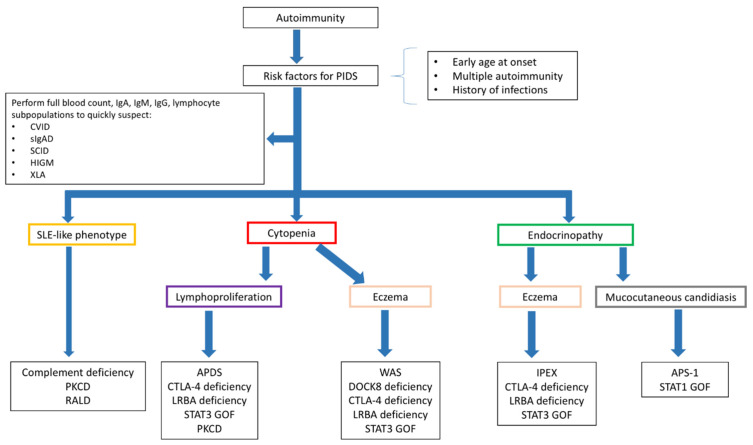
Diagnostic approach to autoimmunity in patients with suspect immunodeficiency. Figure legend: APDS: Activated phosphoinositide 3-kinase d syndrome; APS-1: Autoimmune polyendocrine syndrome 1; CTLA-4: Cytotoxic lymphocyte antigen 4; CVID: Common variable immunodeficiency; HIGM: Hyper-IgM syndrome; LRBA: LPS-responsive beige-like anchor protein; PKCD: protein kinase C δ deficiency; RALD: Ras-associated leukoproliferative disorder; SCID: severe combined immunodeficiency; sIgAD: Selective IgA deficiency; SLE: systemic lupus erythematosus; STAT: Signal Transducers and Activator of Transcription; WAS: Wiskott-Aldrich syndrome; XLA: X-linked agammaglobulinemia.

The diagnostic approach to a child with autoimmune manifestations should include the detailed analysis of the clinical history to evidence the occurrence of infections, quantify their impact, and point out the associated clinical features suggestive for a PID (growth delay, high frequency of infections, need of hospitalization for infections, prolonged use of antibiotics, infections by unusual pathogens, and others). Additionally, a baseline immunological assessment including the determination of serum Ig levels and lymphocyte subpopulations should be performed in all the children with autoimmune manifestations. The need for other specific investigations (Figure 3) varies depending on the specific autoimmune phenotype. The approach to children with immune cytopenia is of particular interest, since it can represent the presentation sign of a wide spectrum of PIDs, but also the first manifestation of a systemic connective tissue disease, such as SLE [120]. As a consequence, the clinical and laboratory assessment of a child with suspected immune cytopenia should comprehend both the analysis of the immune response and the determination of the most relevant autoantibody subclasses, including antinucleous antibodies and antithyroid antibodies [121]. Interestingly, patients with PID have a 120-fold high risk of developing autoimmune cytopenia compared to the general population, with a higher increase in risk observed for AIHA [122]. The case of children presenting with multiple cytopenias is of significant interest. Indeed, studies on Evans syndromes showed that almost half of the children with this condition have e positive genetic testing for PID and that in this cohort of patients the incidence of systemic autoimmune diseases is also considerable [123,124]. In patients with autoimmune endocrinopathy, the suspect of PID should be posed when the disease onset is earlier than usual, when there is an association of two or more endocrine disorders, and when other signs suggestive for PID are present. In this subset of patients, the finding of eczema, elevated serum IgE levels, and peripheral eosinophilia should induce the suspect of a Treg-mediated disorder [125], while the association with chronic mucocutaneous candidiasis is observed in patients with APS-1 and STAT1 GOF [126]. Finally, in the case of SLE-like manifestations, the serum levels of the complement fractions need to be determined [127]. Specific investigations to allow a definitive diagnosis, including the analysis of the immune response to vaccines (for the clinical diagnosis of CVID), extended determination of the lymphocyte subpopulations (including memory B and T cells), functional analysis, and cytogenetic and genetic testing, should be performed based on the clinical suspect.

### 9.2. Diagnosing Autoimmunity in Children with PIDs

Periodic surveillance for autoimmune manifestations is mandatory in all patients with PIDs. This is particularly relevant for children carrying genetic mutations with a well-defined association with autoimmunity (i.e., CTLA-4, LRBA, PI3K mutations). Moreover, there is increasing interest in the identification of potential immunological predictors of autoimmunity in patients with PIDs. Although there are no specific immunological markers with high predictive value for the development of autoimmunity, literature data from patients with primary antibody deficiencies and CID have identified several potential candidates.

A large cohort study on CVID patients with immune cytopenia demonstrated higher levels of serum immunoglobulin, CD19hi B cells, and T CD4 effector T cells, accompanied by reduced naïve T cells [128]. Absence or reduced switched memory B cells have been associated with autoimmune cytopenias, systemic autoimmune diseases, splenomegaly, granulomatous diseases, and lymphadenopathy [129,130]. An expansion of CD21^low^ B cells has been described in association with reduced Tregs in CVID patients with autoimmunity [131]. It has been found that CD21^low^ B cells produce significantly more IgM than naïve B cells after stimulation with CD40L, IL-2, and IL-10 and that CVID patients with autoimmunity have higher levels of IgM compared with non-autoimmune phenotypes [15,132], thus suggesting that increased IgM levels may be a marker of autoimmunity and they may have a pathogenic role. Additionally, lower naïve CD4+ and CD8+ T cells and increased differentiated T cells have been described in CVID patients with autoimmunity [18].

Concerning combined immunodeficiencies, a recent study by Montin et al. in patients with 22q11.2DS highlighted that some immunological features, including a reduced number of naïve T cells, reduced RTE, and elevation of naïve B cells are associated with the development of hematologic autoimmunity and can be evidenced significantly before the onset of autoimmunity [133]. Moreover, the degree of T-cell lymphopenia has been suggested as a contributing factor for the development of autoimmunity in this disease [75]. 

## 10. Conclusions

It is well recognized that autoimmune manifestations are observed in a significant percentage of patients with PIDs, often representing the first sign of these conditions. Patients with early-onset autoimmunity, an association between two or more autoimmune manifestations, or increased susceptibility to infections should be promptly screened for PIDs. Although the intriguing mechanisms underlying the development of autoimmunity in patients with PIDs are far to be completely elucidated, the rapidly evolving knowledge in the genetic background of PIDs will hopefully help to characterize the defects linking immunodeficiency and autoimmunity, thus providing interesting diagnostic and therapeutic implications.

## Figures and Tables

**Table 1:** Autoimmune/inflammatory manifestations in primary antibody deficiencies.

Disease	% of Patients with Autoimmunity	Autoimmune/Inflammatory Manifestations
CVID Specific genetic associations TACI defect BAFF-R defect ICOS deficiency NF-kB1 deficiency NF-kB2 deficiency	20–30%	Autoimmune cytopenias (ITP, AIHA, neutropenia), organ specific autoimmune diseases (e.g., thyroiditis, T1D, ILD, IBD), systemic autoimmune diseases (RA, SLE), lymphoproliferation, lymphoma Variable autoimmune manifestations Variable autoimmune manifestations Autoimmune cytopenias, enteropathy, RA, SLE Autoimmune cytopenias, enteropathy, lymphoproliferation, lymphoma Autoimmunity affecting skin, hair and nails, pituitary hormone deficiencies, autoimmune cytopenias
sIgAD	5–30%	Celiac disease, autoimmune cytopenias (ITP, AIHA), hypothyroidism, Graves’ disease, T1D, RA, SLE.
Hyper IgM syndromes XHIM AID deficiency NEMO	10–20% 21%	AIHA, ITP, autoimmune hepatitis, T1D, Chron’s disease and uveitis, seronegative arthritis, hypothyroidism, SLE, sclerosing cholangitis AIHA, ITP, autoimmune hepatitis, T1D, Chron’s disease and uveitis, lymphoproliferation IBD, arthritis, AIHA
X-linked agammaglobulinemia	15%	Arthritis, DM, IBD, AIHA, scleroderma, alopecia, T1D, glomerulonephritis

ITP, Immune thrombocytopenic purpura; AIHA, autoimmune hemolytic anemia; T1D, Type 1 Diabetes; ILD, interstitial lung disease; IBD, inflammatory bowel disease; RA, rheumatoid arthritis; SLE, systemic lupus erythematosus; NF-kB1, Nuclear factor kappa-light chain enhancer; LRBA, LPS, responsive beige-like anchor protein; CTLA-4, cytotoxic lymphocyte antigen 4; PI3Kδ, Phosphoinositide 3-kinase δ; STAT3, signal transducer and activator of transcription; sIgA, selective immunoglobulin A deficiency; XHIM, X linked variant of hyper-IgM syndrome; AID, activation induce cytidine deaminase; DM: dermatomyositis.

## Data Availability

Not applicable.
